# Prognostic survival biomarkers of tumor-fused dendritic cell vaccine therapy in patients with newly diagnosed glioblastoma

**DOI:** 10.1007/s00262-023-03482-8

**Published:** 2023-06-29

**Authors:** Jun Takei, Yuko Kamata, Toshihide Tanaka, Nei Fukasawa, Kazutaka Gomisawa, Mari Satake, Ryosuke Mori, Yohei Yamamoto, Tomoya Suzuki, Ayaka Oda, Mutsunori Murahashi, Takahiro Fukuda, Masayuki Shimoda, Yuichi Murayama, Yasuharu Akasaki

**Affiliations:** 1 Department of Neurosurgery, The Jikei University School of Medicine, 3-25-8 Nishishinbashi, Minato-Ku, Tokyo, 105-8461 Japan; 2 Division of Oncology, Research Center for Medical Sciences, The Jikei University School of Medicine, Tokyo, Japan; 3 Department of Neurosurgery, The Jikei University Kashiwa Hospital, Chiba, Japan; 4 Department of Pathology, The Jikei University School of Medicine, Tokyo, Japan; 5 Department of Neurosurgery, The Jikei University Daisan Hospital, Tokyo, Japan; 6 Medical Center for Memory and Cognitive Disorders, Sasebo Chuo Hospital, Nagasaki, Japan

**Keywords:** Dendritic cell, Whole tumor cell, Immunotherapy, Glioblastoma, Prognostic factor

## Abstract

Dendritic cell (DC)-based immunotherapy has been applied to glioblastoma (GBM); however, biomarkers informing response remain poorly understood. We conducted a phase I/IIa clinical trial investigating tumor-fused DC (TFDC) immunotherapy following temozolomide-based chemoradiotherapy in patients with newly diagnosed GBM and determined prognostic factors in patients receiving TFDC immunotherapy. Twenty-eight adult patients with GBM isocitrate dehydrogenase (*IDH)* wild-type (*IDH-*WT) were enrolled; 127 TFDC vaccine injections (4.5 ± 2.6 times/patient) were administered. Patients with GBM *IDH-*WT had a respectable 5-year survival rate (24%), verifying the clinical activity of TFDC immunotherapy, particularly against *O*^6^-methylguanine-DNA methyltransferase (MGMT) unmethylated GBM (5-year survival rate: 33%). To identify novel factors influencing overall survival (OS) in GBM *IDH*-WT treated with TFDC immunotherapy, clinical parameters were assessed and comprehensive molecular profiling involving transcriptome and exome analyses was performed. MGMT promoter methylation status, extent of tumor resection, and vaccine parameters (administration frequency, DC and tumor cell numbers, and fusion ratio) were not associated with survival following TFDC immunotherapy. Old age and pre- and post-operative Karnofsky performance status were significantly correlated with OS. Low HLA-A expression and lack of *CCDC88A*, *KRT4, TACC2,* and *TONSL* mutations in tumor cells were correlated with better prognosis. We validated the activity of TFDC immunotherapy against GBM *IDH*-WT, including chemoresistant, MGMT promoter unmethylated cases. The identification of molecular biomarkers predictive of TFDC immunotherapy efficacy in GBM *IDH*-WT will facilitate the design of and patient stratification in a phase-3 trial to maximize treatment benefits.

**Supplementary Information:**

The online version contains supplementary material available at 10.1007/s00262-023-03482-8.

## Introduction

Glioblastoma (GBM) is the most common malignant primary brain tumor, accounting for 56.1% of all gliomas [1]. The median overall survival (OS) of GBM patients following surgical intervention, radiotherapy, and concomitant and adjuvant temozolomide (TMZ) is approximately 14.6 months [2]. Cancer immunotherapy is a broad modality comprising several technologies, including immune checkpoint inhibitors (ICIs), that have emerged as an attractive approach to cancer treatment, particularly in patients with dismal prognosis. Extensive research has been conducted on the application of immunotherapy to treat GBM, with a particular focus on targeting immune checkpoint molecules, cytokines, tumor-associated macrophages (TAMs), and dendritic cells (DCs) [3]. Recently, a phase III immunotherapy clinical trial for GBM treated with DC vaccinations achieved extended patient survival compared with an externally controlled cohort [4].

DCs mediate innate and induce adaptive immune responses [5] and are considered the most potent and versatile antigen-presenting cells, readily initiating T-cell responses [6]. CD8^+^ cytotoxic T lymphocytes require cross-priming by DCs to initiate a productive T-cell response to tumor cells and viruses [7]. DCs are considered promising tools for cancer immunotherapy. DC-based immunotherapy has been described as a potential therapeutic strategy to improve clinical outcomes for GBM patients. Various approaches, including pulsing DCs with glioma tissue or peptides, have been attempted [4, 8]; in the past, we conducted a clinical trial using a unique methodology of the tumor-fused DC (TFDC) [9–12].

To create the TFDC vaccine, patient-derived tumor cells were cultured and expanded. The advantage of using cultured tumor cells for DC activation is that the required amount of tumor-associated antigens (TAAs) can be obtained efficiently, even for small tumor biopsy specimens. In TFDCs, the cytoplasm of DCs and whole-tumor cells is integrated without nuclear fusion, allowing retention of the functions of both cell types, including co-expression of tumor-derived whole TAAs and DC-derived major histocompatibility complex (MHC) class I/II molecules [13]. Generally, TFDCs process various antigenic peptides from whole-tumor cells, which are loaded on MHC class I molecules on the cell surface, not needing to take up exogenous antigens. Subsequently, the antigenic peptide–MHC class I complexes can stimulate CD8^+^ T cells [13]. Thus, TFDC immunotherapy is expected to facilitate more effective antigen presentation than other DC-based immunotherapies.

We have previously described the safety, feasibility, and mechanisms of TFDC therapy, including cytoplasmic accumulation of tumor antigen following TMZ-based chemoradiotherapy as well as the immunological and clinical responses in GBM patients [11]. In our previous phase I/IIa study, 22 patients with newly diagnosed and 10 patients with recurrent GBM underwent TFDC immunotherapy combined with TMZ, with a median OS of 30.5 and 18.0 months, respectively [11]. We only observed transient grade 1 toxicity of injection-site reactions [11]. This clinical trial demonstrated that TFDC immunotherapy is a safe and effective treatment method for patients with GBM and highlighted the need to identify biomarkers that predict patient response to the TFDC vaccine.

Immunomodulatory factors within the tumor microenvironment (TME), including PD-L1 expression, T-cell infiltration, tumor mutational burden (TMB), and HLA expression, have been widely reported to correlate with immunotherapeutic responses [14–18]. However, the biomarkers predicting response to immunotherapy against GBM may differ from those against other cancers. Gromeier et al. [17] reported that a low TMB is associated with favorable clinical outcomes following ICI or oncolytic virus therapy efficacy in patients with malignant gliomas and may therefore represent a novel method to stratify patients for cancer immunotherapy [17]. Additionally, Zhang and colleagues have reported the potency of machine learning algorithms to validate the predictive capacity of immune cell-related long non-coding RNAs for prognosis and immunotherapy response in patients with GBM [19].

In the context of DC-based immunotherapy, a variety of prognostic factors have been investigated, including: conventional factors such as age and resection rate [20, 21]; MGMT methylation status [20–26]; immunological response as assessed by ELISPOT or Tetramer assay [11, 27, 28]; tumor-infiltrating lymphocyte (TIL) presence [28, 29]; and PD-L1 expression levels [25]. However, detailed molecular investigations of tumor cells for the identification of prognostic factors have not been conducted. Moreover, it is unknown whether conventional prognostic factors impact the OS of patients treated with TFDC-based immunotherapy.

Therefore, the primary aim of the current study was to explore novel factors predicting the response to TFDC-based immunotherapy with TMZ in patients with GBM, via comprehensive molecular profiling analyses.

## Materials and methods

### Patients

**Fig. 1:**
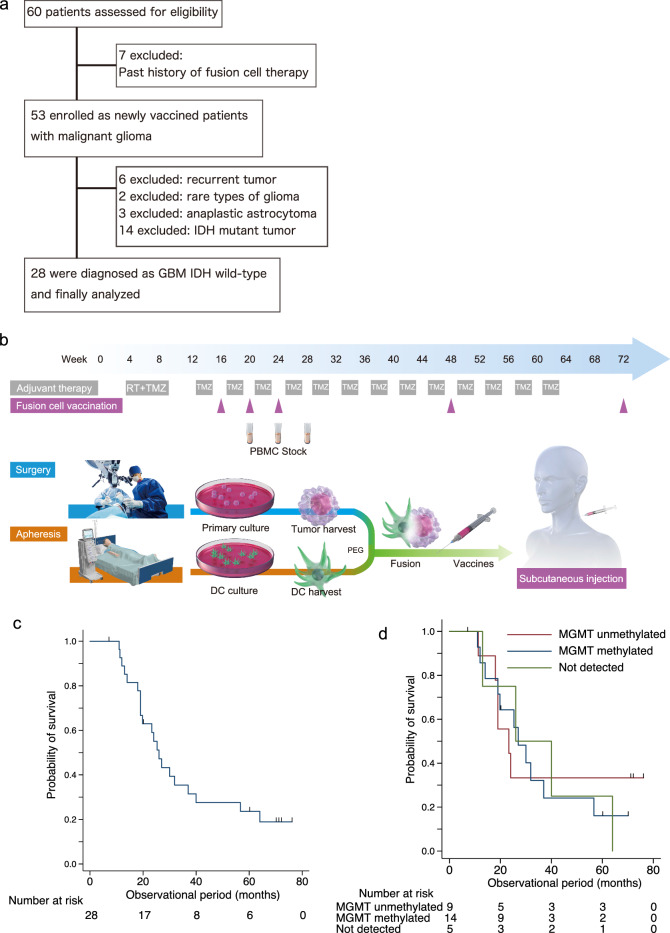
Schematic diagram of the present study. **a** Flowchart of patients included in the present study. **b** Tumor cells from surgical specimens and DCs purified from PBMCs were fused, and three doses of the vaccine plus one to two booster doses were administered following postoperative chemoradiotherapy. **c** Kaplan–Meier analysis of survival probability in the current cohort (*n* = 28). **d** Kaplan–Meier analysis of survival probability in GBM *IDH* wild-type tumors stratified by MGMT promoter methylation status (*n* = 28)

Inclusion and exclusion criteria for this study were the same as previously described [11]. In the current study, patients with GBM IDH-WT were eligible. Sixty patients who underwent TFDC vaccination from January 2006 to December 2016 were enrolled; of these, seven with a history of TFDC vaccination were excluded. Subsequently, 53 patients who were newly vaccinated with TFDC vaccines were screened, and three blinded pathologists (NF, KG, and MS) diagnosed all surgical specimens based on the World Health Organization (WHO) 2016 classification. Twenty-five patients were excluded because of the presence of a recurrent tumor (*n* = 6), rare type of tumor according to pathological re-diagnosis (*n* = 2), diagnosis of anaplastic astrocytoma (*n* = 3), or IDH mutant tumors (*n* = 14). Thus, 28 eligible patients, aged 21–74 years (median: 52 years), were enrolled and received a total of 127 vaccine injections (Fig. 1a). Of these, 19 patients were included in a previous study [11]. In the current study, we performed data fixation on December 31, 2020, with a follow-up period of 32.8 ± 21.8 (mean ± standard deviation (SD)) months.

### TFDC vaccination

TFDC vaccines were generated from cultured tumor cells derived from surgical specimens and DCs from peripheral blood, as described previously [11, 12]. The vaccine was subcutaneously administered in the cervical region, according to our protocol, as shown in Fig. 1b.

### Immunohistochemical analyses

For immunohistochemical staining, 4-μm sections of formalin-fixed paraffin-embedded (FFPE) tissues obtained from the 45 patients were incubated with anti-ATRX (1:500, HPA001906; Sigma-Aldrich, St. Louis, MO, USA) and anti-p53 (clone DO-7, mouse, prediluted; Nichirei Biosciences Inc, Tokyo, Japan) antibodies, according to the manufacturers’ protocols. All slides were reviewed by three blinded pathologists (NF, KG, and MS). ATRX and p53 expression was assessed according to the cIMPACT-NOW update 2 [30].

In 28 GBMs, additional immunohistochemistry was performed with anti-HLA-A (1:3,000, ab52922; Abcam, Cambridge, UK), anti-CD3 (1:100, ab5690; Abcam), anti-CD8 (1:100, ab17147; Abcam), anti-FoxP3 (1:100, D2W8E, #98,377; Cell Signaling Technology, Danvers, MA, USA), anti-PD-1 (1:200, D4W2J, #86,163; Cell Signaling Technology), and anti-PD-L1 (1:200, E1L3N, #13,684; Cell Signaling Technology) antibodies. Antigen retrieval was performed in citrate buffer, pH 6.0 (ATRX, CD3, CD8, and Foxp3) or EDTA buffer, pH 9.0 (PD-1 and PD-L1) in an autoclave. Tonsil tissue was used as a control material for HLA-A, CD3, CD8, Foxp3, PD-1, and PD-L1 staining.

To assess PD-1, CD3, CD8, and Foxp3 expression, the stained sections were surveyed under a low-power field (× 40), and five hot spots were selected. Positive cells in these areas were counted in a high-power field (× 400, 0.47 mm^2^) [31]. For quantitative evaluation of HLA-A, the stained sections were screened in a low-power field (× 40), and a middle-power field (× 200) with the densest spot was assessed. HLA-A positive areas were determined using Fiji software (version 2.0.0-re-69/1.52p) [32]. Briefly, a brown channel was extracted from the image using “color deconvolution” and “H DAB” functions. The brown channel image can be thresholded from 30 to 150 and measured as HLA-A-positive areas. PD-L1 expression was scored as a percentage of tumor cells expressing PD-L1: 3 + , ≥ 50%; 2 + , ≥ 5% and < 50%; 1 + , ≥ 1% and < 5%, and 0, < 1% [31]. This assessment was performed by JT and NF in a blinded manner.

### Nucleic acid extraction from paraffin-embedded tissues and tumor cells

Two 10-μm FFPE sections were used for DNA extraction with the Allprep DNA/RNA FFPE kit (QIAGEN, Venlo, the Netherlands). Cryopreserved tumor cells and patient-derived PBMCs were thawed immediately before use. DNA and RNA were extracted from subconfluently grown tumor cells and PBMCs using the DNeasy Blood & Tissue kit and RNeasy plus mini kit or Allprep DNA/RNA mini kit (QIAGEN), respectively. Prior to use, the extracted DNA and RNA were stored in –20 °C and –80 °C freezers, respectively.

## Detection of *IDH1/2* mutations

To detect the R132H point mutation at codon 132 of *IDH1*, a Cycleave RT-PCR [33] was performed on DNA from all 45 tumors. Briefly, genomic DNA was extracted, and *IDH1* was amplified using real-time PCR on a QuantStudio 5 RT-PCR system (Applied Biosystems, Waltham, MA, USA) using the CycleavePCR mix (TaKaRa Bio Inc, Kusatsu, Japan) with R132H-specific and wild-type probes. The primer set and probe sequences were as follows: forward primer, 5′-ACCAAATGGCACCATACGA-3′; reverse primer, 5′-TCATACCTTGCTTAATGGGTGT-3′; wild-type probe, 5′-Eclipse-ataggtcgtc-HEX-3′; and R132H mutation probe, 5′-Eclipse-gat*g*acctatg-FAM-3′.

Sanger sequencing for *IDH1* codon 132 and *IDH2* codon 172 mutations was performed using the following primer sets: *IDH1* forward, 5′-ACCAAATGGCACCATACGA-3′; *IDH1* reverse, 5′-TCATACCTTGCTTAATGGGTGT-3′; *IDH2* forward, 5′-GCTGCAGTGGGACCACTATT-3′; *IDH2* reverse, 5′-CAAGAGGATGGCTAGGCGAG-3′. The following conditions were used for DNA amplification: 35 cycles of denaturation (98 °C for 10 s), primer annealing (60 °C for 5 s), and extension (68 °C for 1 s). Finally, direct sequencing was performed using a 3730xL DNA analyzer (Applied Biosystems) with the BigDye® Terminator v3.1 cycle sequencing kit (Applied Biosystems). The *IDH1* codon 132 and *IDH2* codon 172 wild-type sequences used for reference were AGGTCGTC and TGGCAGGCAC, respectively [34].

### Methylation-specific PCR for detecting *MGMT* promoter methylation

A previously reported protocol [35] was slightly modified to detect DNA in FFPE sections. Briefly, DNA was treated with bisulfite using the EZ DNA Methylation-Gold kit (Zymo Research, Irvine, CA, USA). The primer sequences for *MGMT* [35] were forward: 5′-GAGAGATTTGTGTTTTGGGTTTAGTG-3′ and reverse: 5′-CCTTCAACCAATACAAACCAAACAA-3′. PCR was performed using 50–100 ng of bisulfited DNA with the AmpliTaq Gold™ 360 master mix (Applied Biosystems) as follows: denaturation at 94 °C for 10 min, followed by 35 cycles of denaturation (94 °C for 30 s), primer annealing (62 °C for 30 s), and extension (72 °C for 30 s), and a final extension at 72 °C for 10 min. CpGenome Universal Methylated DNA (Merck Millipore, Burlington, MA, USA) and CpGenome Universal Unmethylated DNA (Merck Millipore) were used as positive and negative controls. PCR products (10 μL) were analyzed on 2% agarose gels stained with ethidium bromide at a final concentration of 0.1 μg/mL. If neither band was detected, the result was recorded as “not detected.”

### Whole-transcriptome sequencing and analysis

For RNA quality control, the RNA integrity number (RIN) was determined using the Agilent 2100 bioanalyzer (Agilent Technologies, Santa Clara, CA, USA). Samples with RIN values ≥ 8.6 were used for library preparation. Poly(A) RNA was extracted from 2 μg of total RNA using the Dynabeads mRNA DIRECT micro kit (Thermo Fisher Scientific, Waltham, MA, USA). Preparation of RNA libraries using the Ion Total RNA-Seq kit v2 (Thermo Fisher Scientific) and sequencing on Ion Chef and Ion Proton systems (Thermo Fisher Scientific) were performed.

All RNA sequencing data were processed using the CLC Genomics Workbench (QIAGEN), and the reads were mapped to the ENSEMBL reference human genome GRCh37. All samples were divided into two groups based on patient median OS and analyzed for differences in gene expression; genes with a fold change > 2 and *p* < 0.05 were considered to be significantly differentially expressed. Gene Ontology (GO) analysis was performed using the GO Consortium resources (http://geneontology.org/). Genes included in a specific GO term were assigned to two groups based on the median expression levels to determine differences in survival between the high- and low-expression groups. Based on the median expression values of *HLA-A*, *HLA-B*, *HLA-C*, *HLA-DPA*, *HLA-DQA*, and *HLA-DRA*, our cohort and the GBM cohort from the Chinese Glioma Genome Atlas (CGGA) dataset, obtained from GlioVis [36] (http://gliovis.bioinfo.cnio.es/), were divided into high- (top 50%) and low- (bottom 50%) expression groups. Search settings were as follows: Dataset: Adult and CCGA, Tumor type: Primary, Gene: *HLA-A*, *HLA-B*, and *HLA-C*, Histology: GBM, Subtype: All, Gender: All, IDH status: Wild-type, Cutoff: Median. The CGGA dataset included 220 patients with the GBM *IDH* wild-type.

### Reverse transcription (RT)-quantitative PCR

For mRNA expression analysis, total RNA from cultured tumor cells was reverse-transcribed using the PrimeScript RT master mix (TaKaRa Bio, Inc.). Real-time amplification was achieved using a QuantStudio 5 RT-PCR system (Applied Biosystems); three biological replicates were used. mRNA expression was analyzed using TaqMan gene expression assays (Applied Biosystems), with Hs02786624_g1 for *GAPDH* used as an internal control and Hs01058806_g1 for *HLA-A* as a target gene. PCR was performed at the following conditions: denaturation at 95 °C for 10 min, followed by 40 cycles at 95 °C for 15 s and 60 °C for 1 min. *HLA-A* mRNA expression was calculated using the 2^−ΔΔCt^ method. One case was used as a reference to evaluate the relative expression levels.

### Whole-exome sequencing

Tumor DNA and genomic DNA derived from PBMCs were used for whole-exome sequencing. Exome libraries were prepared using the Ion AmpliSeq Exome RDY kit (Thermo Fisher Scientific). Sequencing was performed on Ion Chef and Ion Proton systems (Thermo Fisher Scientific). All exon sequencing data were processed using the CLC Genomics Workbench 21.0.5 (QIAGEN), and the reads were mapped to the ENSEMBL reference human genome GRCh37. The oncoplot was constructed with R 4.1.2, Rstudio v2021.09.01 + 372, and Maftools 2.10.0 [37] following conversion of variant calling files to mutation annotation format files with vcf2maf v1.6.21 [38], Ensemble variant effect predictor 104.3 [39], and Miniconda. TMB was defined as the number of somatic nonsynonymous mutations per megabase in the target region of the exome panel.

### Statistical analysis

Continuous data are expressed as the mean ± SD, and categorical data are expressed as numbers and percentages. KPS is expressed as the median and interquartile range (IQR). To compare characteristics among patients in subgroups with different *MGMT* promoter methylation status, such as unmethylated, methylated, and not detected, the Kruskal–Wallis rank test and Fisher’s exact test were used, as appropriate.

OS was calculated from the day of the initial surgery until the date of death due to any cause or until censored. The log-rank test was used to compare survival differences for each variable. Univariate and multivariate Cox proportional regression analyses were used to assess the association between OS and other variables. For evaluation of vaccine parameters, OS was defined from the day of the third vaccination or last vaccination, if participants received vaccines fewer than three times until the date of death owing to any cause. Multivariate analyses were performed on parameters that were estimated to be relevant by a consensus of the clinical team and statistical experts. Cases with missing data were omitted, and the remaining available data were analyzed. Statistical analyses were performed using STATA 14 (StataCorp LLC, College Station, TX, USA) or GraphPad Prism version 9 (GraphPad Software, Boston, MA, USA). All *p*-values were two-sided, and the significance level was set at *p* < 0.05.

## Results

### Association between *MGMT* promoter methylation status and patient OS

The demographic data for all patients are presented in Table 1. The median survival times and 5-year survival rates of GBM *IDH-WT* were 26.0 months and 23.2%, respectively (Fig. 1c, Table 1). MGMT promoter methylation is an independent favorable prognostic factor in patients with GBM who receive TMZ and radiotherapy [40]. The 28 *IDH-WT* GBM tumors were stratified into three subgroups based on *MGMT* promoter methylation status (Fig. 1d). Kaplan–Meier analysis showed that the 5-year survival rates in the subgroups with unmethylated, methylated, and undetected *MGMT* were 33.3%, 16.7%, and 25.0%, respectively (Fig. 1d). The 2-year survival of patients with GBM with unmethylated MGMT is typically less than 10% [40]; thus, this excellent survival rate demonstrated the effectiveness of TFDC therapy for this typically chemoresistant subgroup of GBM. The patients with unmethylated *MGMT* in their tumors survived longer than those from the other two subgroups; however, no significant difference was detected compared with the methylated group and not-detected group (*p* = 0.814 and *p* = 0.738, respectively; Fig. 1d). The univariate Cox proportional hazards regression model determined that *MGMT* promoter methylation status was not a prognostic factor for patients with *IDH-WT* GBM (Table 2), whereas age and pre-/postoperative KPS were. After age and sex adjustments, pre-/postoperative KPS was not a prognostic factor (Table 2). TFDC-based vaccine parameters were not significant prognostic factors (Supplementary Table S1).

**Table 1:** Baseline characteristics of patients who underwent TFDCs therapy in this study

	IDH wild-type	IDH mutant	Total	*p*-value
Numbers	31	14	45	
Sex				p = 0.744*
Female	11	6	17	
Male	20	8	28	
Age–years				*p* = 0.004†
Mean ± SD	54.9 ± 16.0	42.2 ± 7.1	50.9 ± 14.9	
Pathology				
Glioblastoma	28	2	30	
Anaplastic astrocytoma	3	7	10	
Anaplastic oligodendroglioma	0	4	5	
Oligodendroglioma	0	1	1	
Extent of resection				*p* = 0.592*
Total	16	8	24	
Subtotal	5	4	9	
Partial	8	2	10	
Biopsy	2	0	2	
MGMT profile (MS-PCR)				*p* = 0.750*
Methylated	16	9	27	
Unmethylated	9	3	12	
Not detected	6	2	8	
Bevacizumab usage				*p* = 0.753*
Yes	13	5	18	
no	18	9	27	
Preo*p*erative KPS				*p* = 0.048†
Median	80	100	90	
IQR	70–100	90–100	70–100	
Postoperative KPS				*p* = 0.016†
Median	90	100	90	
IQR	70–100	90–100	80–100	
RPA2011 classification				*p* = 0.001*
III	4	9	13	
IV	21	5	26	
V	6	0	6	
Overall survival –months				*p* = 0.002††
Median	27	not reached	40	
5 year survival rate(%)	28.1%	78.6%	44.5%	
Number of injections				*p* = 0.248†
Total	142	74	216	
Mean ± SD	4.6 ± 2.5	5.3 ± 2.4	4.8 ± 2.5	
PBMC cell numbers				*p* = 0.085†
Mean	1.0*10^8^	6.5*10^7^	9.0*10^7^	
SD	1.1*10^8^	5.6*10^7^	9.4*10^7^	
Dendritic cell numbers				*p* = 0.888†
Mean	4.6*10^6^	3.8*10^6^	4.3*10^6^	
SD	5.0*10^6^	2.4*10^6^	4.3*10^6^	
Tumor cell numbers				*p* = 0.001†
Mean	1.0*10^6^	1.3*10^6^	1.1*10^6^	
SD	1.1*10^6^	0.9*10^6^	1.0*10^6^	
Fusion ratio (Dendritic cell/Tumor cell)				*p* < 0.001†
Mean ± SD	8.8 ± 14.9	5.3 ± 6.5	7.7 ± 12.4	
Dendritic cell generating ratio (%)				*p* = 0.314†
Mean ± SD	8.1 ± 4.9	8.3 ± 4.1	8.1 ± 4.6	

*Fisher’s exact test, †Mann–Whitney U test IQR: interquartile range, KPS: Karnofsky performance status

^††^log-rank test, MGMT: O6-methylguanine-DNA methyltransferase, MS-PCR: methylation specific-polymerase chain reaction, RPA: recursive partitioning analysis, SD: standard deviation, TFDC: tumor-fused dendritic cells

**Table 2:** Cox regression analysis for overall survival in patients with malignant glioma treated with TFDCs immunotherapy

Univariate				Multivariate			
independent variables	Hazard ratio	95% Confidence interval	*p* value	Age and sex adjusted	Hazard ratio	95% Confidence interval	*p* value
Age (per 10 years)	1.63	1.23 to 2.16	0.001	Age (per 10 years)	1.51	1.14 to 2.01	0.004
Sex (Female)	1.37	0.63 to 3.01	0.422	Sex (Female)	2.13	0.94 to 4.84	0.069
Extent of resection				WHO grade IV	7.09	2.02 to 25.0	0.002
Not total resection	1	Reference					
Total resection	0.78	0.36 to 1.68	0.521	Age (per 10 years)	1.51	1.16 to 1.98	0.002
WHO grade				Sex (Female)	2.39	1.04 to 5.48	0.040
II		omitted		IDH mutant	0.22	0.07 to 0.71	0.011
III	1	Reference					
IV	8.48	2.47 to 29.1	0.001	Age (per 10 years)	1.60	1.20 to 2.14	0.001
IDH genetic status				Sex (Female)	1.86	0.84 to 4.13	0.124
IDH wild-type	1	Reference		Preoperative KPS (per 10score)	0.83	0.66 to 1.05	0.129
IDH mutant	0.21	0.07 to 0.62	0.005				
MGMT promoter methylation				Age (per 10 years)			
(MS-PCR)					1.59	1.18 to 2.15	0.002
Unmethylated	1	Reference		Sex (Female)	1.79	0.80 to 3.99	0.158
Methylated	0.81	0.32 to 2.04	0.660	Postoperative KPS (per 10score)	0.76	0.55 to 1.05	0.092
Not detected	1.18	0.39 to 3.53	0.766				
Preoperative KPS(per 10score)	0.82	0.68 to 0.99	0.041	Age (per 10 years)	1.64	1.21 to 2.22	0.001
Postoperative KPS(per 10score)	0.66	0.49 to 0.90	0.008	Sex (Female)	1.95	0.86 to 4.43	0.111
RPA2011 classification				RPA2011 classification V	1.57	0.57 to 4.35	0.385
III	1	Reference					
IV	3.78	1.22 to 11.7	0.022				
V	6.96	1.84 to 26.3	0.004				

IDH: isocitrate dehydrogenase, KPS: Karnofsky performance status, MGMT: O6-methylguanine-DNA methyltransferase, MS-PCR: methylation specific-polymerase chain reaction, RPA: recursive partitioning analysis, TFDC: tumor-fused dendritic cells, WHO: World Health Organization

### Low tumor HLA-A expression predicted better OS in GBMs treated with TFDC immunotherapy

**Fig. 2:**
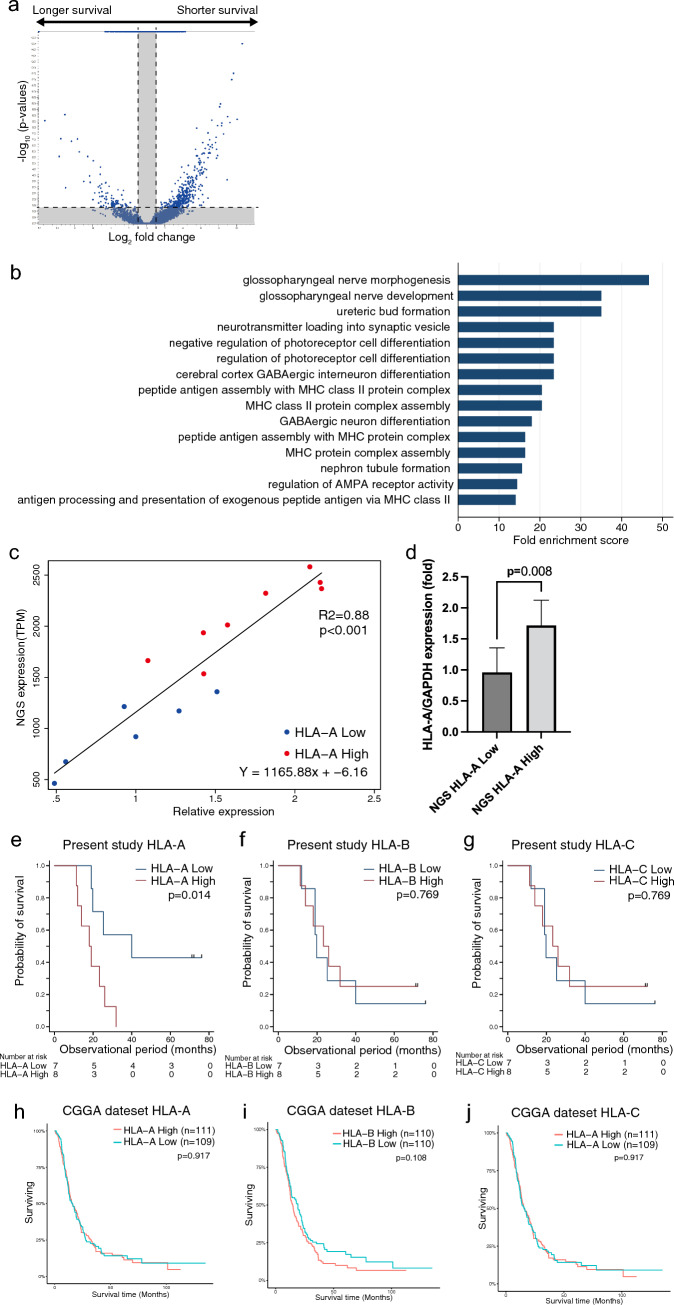
Whole-transcriptome analysis and OS of patients stratified by *HLA* expression levels. Whole-transcriptome analysis was performed on 15 samples derived from GBM *IDH* wild-type. **a** Volcano plot of the distribution of DEGs in patients with longer and shorter survival. **b** Top 15 biological process GO terms associated with 473 DEGs. **c**, **d** RT-PCR analysis was performed for 14 samples derived from GBM *IDH* wild-type. Scatter plot of HLA-A expression levels, determined using NGS and RT-PCR. Blue circles represent the HLA-A low-expression group, and red circles represent the high-expression group (**c**). HLA-A mRNA expression levels were compared between high and low HLA-A expression groups, determined using NGS (**d**). **e**–**g** Kaplan–Meier survival curves for patients with high and low expression levels of *HLA-A* (**e**), *HLA-B* (**f**), and *HLA-C* (**g**) in the study cohort. **h**–**j** Survival curves for patients with high and low expression levels of *HLA-A* (**h**), *HLA-B* (**i**), and *HLA-C* (**j**) in the CGGA dataset from 220 patients with GBM *IDH* wild-type

RNA sequencing was performed and analyzed in 15 of 28 GBM *IDH-WT* specimens. We divided specimens into two groups based on median survival time, and Kaplan–Meier analysis revealed a difference in survival between the groups (Supplementary Fig. S1). There was no significant difference in baseline characteristics between the groups (Supplementary Table S2). In total, 473 differentially expressed genes (DEGs) were identified between the groups (Fig. 2a). GO analysis for biological processes revealed 327 enriched GO terms. Among the 15 GO terms with the highest enrichment scores, 5 were associated with the MHC (Fig. 2b).

To validate the gene expression data with next-generation sequencing (NGS), we used 14/15 tumor samples (one sample was not available because of the lack of RNA sample) to perform RT-PCR analysis of *HLA-A* expression. We confirmed that *HLA-A* expression levels detected via NGS and RT-PCR showed a strong positive correlation (*p* < 0.001; Fig. 2c). Twenty-eight GBM *IDH-WT* tumors were divided into the high- and low-*HLA-A* groups, as determined by NGS, with the mean relative expression levels of *HLA-A* being 1.72 ± 0.40 and 0.96 ± 0.41, respectively (*p* = 0.008; Fig. 2d).

We next investigated the relationship between the MHC and clinical outcomes using the Cox regression model and Kaplan–Meier log-rank test (Fig. 2e–g). Low tumor expression of *HLA-A*, but not *HLA-B* or *HLA-C*, was significantly associated with favorable OS prognosis in GBM patients treated with TFDC immunotherapy (*p* = 0.014). This survival impact of low *HLA-A* was considered specific to TFDC immunotherapy, as analysis of the CGGA GBM dataset revealed no association of *HLA-A*, *HLA-B*, and *HLA-C* with survival (Fig. 2h–j). Additionally, there were no significant associations between OS and *HLA-DPA*, *HLA-DQA*, or *HLA-DRA* levels (Supplementary Fig. S2). Cox regression analysis confirmed that low *HLA-A* expression was the only prognostic factor for survival in this cohort (Supplementary Table S3).

### Analysis of expression of HLA-A and TIL markers and OS

**Fig. 3:**
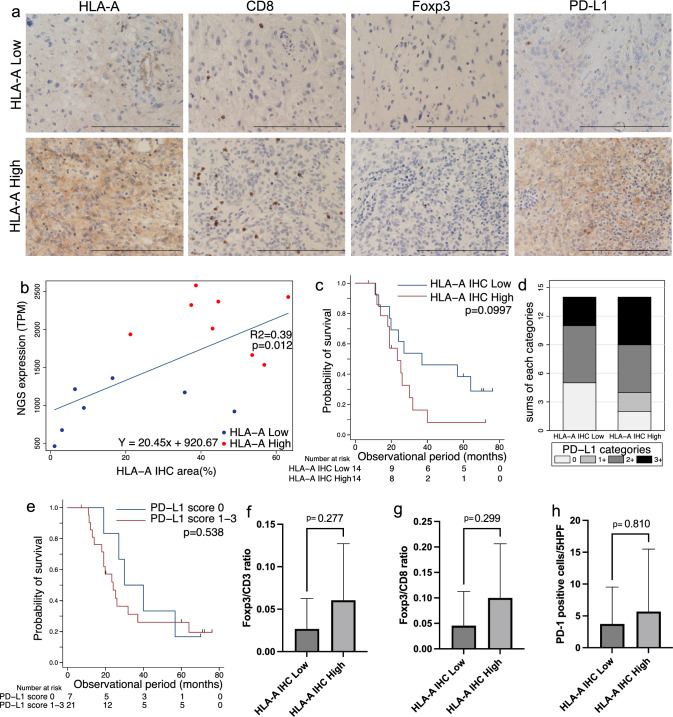
Immunohistochemical analysis of HLA expression in tumor cells and immunoregulatory cell infiltration in the tumor. Immunohistochemical analysis of surgical specimens was performed on 28 samples of GBM *IDH* wild-type. **a** Expression of HLA-A, CD8, Foxp3, and PD-L1 in tumor specimens with high and low *HLA-A* expression levels determined through NGS (×400; bar = 200 µm). **b** Scatter plot of HLA-A expression levels, determined using NGS and IHC-positive areas (*n* = 15). **c** Kaplan–Meier survival curves for patients with high and low HLA-A IHC-positive areas (*n* = 28). **d** PD-L1 positivity in the high and low HLA-A staining groups (*N* = 28). **e** Kaplan–Meier survival curves for patients with expression score of PD-L1 (*n* = 28). **f**, **g** Foxp3/CD3- and Foxp3/CD8-positive cell ratios in the high and low HLA-A staining groups (*n* = 28). Data are presented as the mean ± standard deviation (SD). **h** Numbers of PD-1-positive cells in the high- and low-HLA-A staining groups (*n* = 28). Data are presented as the mean ± SD

We next used immunohistochemical staining of GBM *IDH-WT* surgical specimens (*n* = 28) to assess HLA-A expression and various TIL and immune markers, comparing the HLA-A high and low groups (Fig. 3a). *HLA-A* expression levels detected by NGS and IHC showed a positive correlation (*p* = 0.012; Fig. 3b). We divided 28 GBM cohorts into two groups according to median HLA-A IHC-positive areas. Patients in the HLA-A low-staining group tended to live longer than those in the high-staining group, according to the Kaplan–Meier analysis (Fig. 3c).

The percentage of PD-L1 negative tumors (score 0) was 35.7% and 14.3% in HLA-A low- and high-staining groups, respectively, revealing no significant difference between the groups (Fig. 3d). Although there was no statistical significance, the survival curve of PD-L1-negative tumors (*n* = 7) shifted right compared with that of PD-L1-positive tumors (Fig. 3e).

The mean ratios of Foxp3/CD3 and Foxp3/CD8 in HLA-A high- versus low-staining groups were 0.060 ± 0.067 versus 0.027 ± 0.036, and 0.100 ± 0.106 versus 0.046 ± 0.067, respectively. The Foxp3/CD3 and Foxp3/CD8 ratios were approximately two-fold higher in the high HLA-A staining group than in the low HLA-A staining group; however, the differences were not significant (Fig. 3f, g). There were 5.7 ± 9.8 versus 3.7 ± 5.8 tumor-infiltrating PD-1-positive cells in HLA-A high- versus low-staining groups, respectively, with no significant difference between the groups (Fig. 3h).

### Whole-exome analysis of GBM tumors identified prognostic gene variants in GBM IDH-WT treated with immunotherapy

**Fig. 4:**
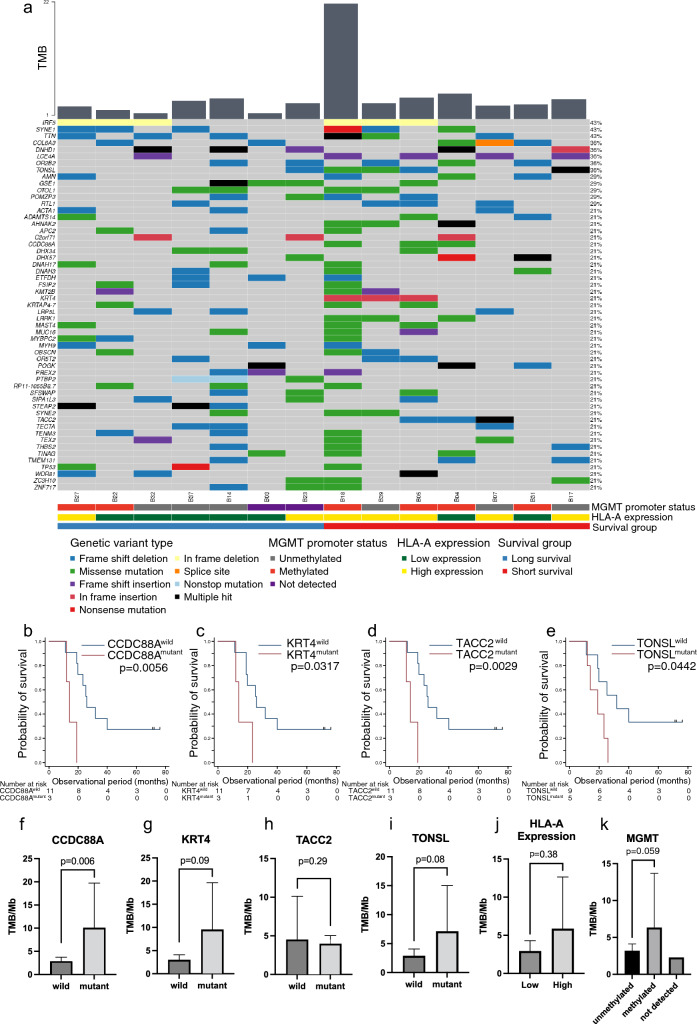
Genetic variations found in 14 GBM tumors using whole-exome sequencing. **a** Whole-exome sequencing was performed on 14 paired samples of PBMCs and tumor DNA derived from GBM *IDH* wild-type. Bar plots show the TMB (number of mutations per megabase) (upper). A total of 55 genetic variants were identified in more than three samples each (middle). The color bar shows MGMT promoter methylation status (gray, unmethylated; orange, methylated; purple, not detected) (upper), sample groups stratified by HLA-A expression (green represents the low-expression group and yellow represents the high-expression group) (middle), and median overall survival (blue represents the long-survival group and red represents the short-survival group) (lower). **b**–**i** Four candidate genes with prognostic mutations and comparison of TMB between wild-type and mutant tumors. **b**–**e** Kaplan–Meier survival curves using the genetic variants to stratify the patient cohort (*n* = 14). **f**–**i** TMB comparisons between the mutant and wild-type tumors for the four identified genes (*n* = 14). **j** TMB comparisons between the high- and low-HLA-A expression groups (*n* = 14). **k** TMB comparisons between MGMT promoter unmethylated (*n* = 7), methylated (*n* = 5), and not detected (*n* = 2) groups. All *p*-values were calculated using the Mann–Whitney *U*-test

We performed whole-exome analysis using DNA from 14/28 GBM *IDH-WT* tumor specimens with matched PBMCs. All exome-seq cases (*n* = 14) were included in RNAseq cases (*n* = 15). Matched PBMCs were not obtained in one case. Figure 4a shows 55 genetic variants, each identified in more than three samples. The median TMB was 3.2 somatic variants per megabase of the target region of the exome panel (IQR: 2.6–4.0). One sample (B18) had a high TMB (21.2 variants/Mb), with 34/55 genetic variants. Most genetic variants were frameshift deletions or missense mutations (Supplementary Fig. S3a, b). Cox regression analysis revealed that higher TMB tended to correlate with a poor prognosis with a hazard ratio (HR) of 1.16 (95% confidence interval [CI]: 0.99–1.35, *p* = 0.054; Supplementary Fig. S3c).

The Cox proportional hazards regression model identified eight candidate genes with variants indicative of survival with *p* < 0.10, namely, *AHNAK2*, *CCDC88A*, *KRT4*, *KRTAP4-7*, *LRRK1*, *STEAP2*, *TACC2*, and *TONSL* (Supplementary Table S4). The *CCDC88A* variants were found in three tumors, all in the short-survival group. *TONSL* variants were observed in 5/14 (36%) patients, all of which belonged to the high *HLA-A* expression group, as determined by NGS; the presence of a *TONSL* mutation was significantly associated with high *HLA-A* expression in tumor cells (Fig. 4a; Supplementary Table S5).

We next attempted to stratify survival using the eight genes and dividing the cohort into two groups based on the presence of variants. Kaplan–Meier analyses and the log-rank test revealed that *CCDC88A*, *KRT4*, *TACC2*, and *TONSL* variants significantly impacted poor prognosis (Fig. 4b–e; Supplementary Table S6).

We examined whether these gene variants were associated with TMB. *CCDC88A* mutant tumors had a significantly higher TMB than did *CCDC88A* wild-type tumors (Fig. 4f). *KRT4* and *TONSL* mutant tumors tended to have an increased TMB compared with their wild-type counterparts (Fig. 4g, i); however, *TACC2* mutant tumors did not (Fig. 4h). The mean TMB was approximately twofold higher in the high *HLA-A* expression group than in the low HLA-A expression group; however, this difference was not significant (Fig. 4j).

All three long-term survivors with GBM *IDH-WT* who lived over 5 years (B14, B32, and B37) were MGMT unmethylated, had low *HLA-A* expression, and had no variants in any of the four prognostic genes, *CCDC88A*, *KRT4*, *TACC2*, and *TONSL* (Figs. 1d, 4a). We, therefore, analyzed whether MGMT promoter methylation status was associated with HLA-A expression, TME features, the TMB, and genetic mutations, which were considered prognostic factors in this study. The HLA-A-positive area and Foxp3/CD8 ratio did not differ in the methylated and unmethylated groups. The Foxp3/CD3 ratio was twofold higher in the methylated group than in the unmethylated group (Supplementary Fig. S4a–c). The number of PD-1-positive cells was tenfold higher in the unmethylated group; however, this difference was not significant (Supplementary Fig. S4d). The mean TMB was approximately two-fold higher in the methylated group than in the unmethylated group, but this difference was not significant (Fig. 4k). *CCDC88A* variants were only found in the methylated group (Fig. 4a, Supplementary Table S7).

## Discussion

We have previously described the safety, effectiveness, and mechanisms of TFDC therapy combined with TMZ, demonstrating immunological and clinical responses in patients with newly diagnosed and recurrent GBM in a phase I/IIa trial [11]. One of the rate-limiting factors in TFDC-based immunotherapy was to secure constant production of the number and viability of harvested tumor cells. The median number of tumor cells in the TFDC immunotherapy used in this study was 0.6 × 10^6^. We previously reported that the efficiency of creating TFDCs is 61.6% with a 2:1 fusion ratio and only 7.2% at a 10:1 ratio [11]. Dhodapkar et al. found that 1.6 to 4.0 × 10^6^ antigen-pulsed DCs induced an effective immune response in healthy adults [41]. Accordingly, we propose a DC count of 1.2 × 10^6^, tumor cell count of 0.6 × 10^6^, and fusion ratio of 2:1 as appropriate conditions for a TFDC vaccine.

There are no biomarkers that can predict the long-term survival of patients with GBM after DC-based immunotherapy. The current study suggested that the level of HLA-A expression in GBM *IDH-WT* was a significant prognostic factor in patients treated with TFDC immunotherapy. Given that T cells in the localized tumor tissue as well as systemic peripheral blood play a pivotal and major role in regulation for anti-tumor immunity, we observed a trend of Foxp3/CD3 and Foxp3/CD8 ratios being higher in the high *HLA-A* expression group than in the low HLA-A expression group. These results suggest that immunosuppressive TILs are more prevalent in tumors with high HLA-A expression. HLA-A expression in the tumor can influence the antitumor immune balance, which may impact tumor response to TFDC immunotherapy. Limited data are available on the impact of HLA expression on the prognosis for GBM. Schaafsma et al. reported favorable outcomes in patients with glioma with low HLA expression who were treated with ICIs; however, this was not observed in those with GBM [18]. In general, tumors with higher levels of HLA-A expression are regarded as immunologically “hot” and good responders to cancer immunotherapy. However, the present study demonstrated that GBMs with lower HLA-A expression were associated with prolonged survival. GBM with downregulated HLA-A expression is considered to be in a particularly immunotherapy-naïve state. Modification of DCs, following stimulation and appropriate antigen presentation, maintains an immunosupportive TME [18, 42], and TFDC-based immunotherapy could provide a clinical benefit to immunologically naïve tumors because of the preferable immunological balance in HLA-A low-expression GBM.

To the best of our knowledge, this is the first study analyzing genetic mutations in GBM patients treated with TFDC immunotherapy to evaluate the effects of gene variants on clinical outcomes. We identified mutations in *CCDC88A*, *KRT4*, *TACC2*, and *TONSL* as potential biomarkers for poor prognosis in GBM patients receiving TFDC-based immunotherapy. Patients with *CCDC88A*, *KRT4*, and *TONSL* mutant tumors tended to have a significantly higher TMB than those with wild-type tumors. Gromeier et al. [17] reported that a very low TMB correlated with longer survival of patients with recurrent GBM following treatment with an oncolytic virus or ICI, as evidenced by immunological engagement. Moreover, the Kyoto Encyclopedia of Genes and Genomes antigen processing and presentation score was significantly higher in non-hypermutational samples (< 10 mutations/megabase) in *IDH-WT* gliomas [43]. Thus, particularly in *IDH-WT* tumors, a low TMB may be associated with a better immunotherapeutic effect. The current study suggests that TMB as well as mutations in *CCDC88A*, *KRT4*, *TACC2,* and *TONSL* could represent important prognostic factors in patients with newly diagnosed *IDH-WT* GBM treated with TFDC immunotherapy. However, the mechanism by which these gene mutations negatively impact prognosis remains unclear.

In the current study, the 5-year survival of patients with GBM with unmethylated MGMT was 33.3%; this excellent survival rate demonstrated the effectiveness of TFDC therapy. However, whether MGMT status is a prognostic factor for GBM treated with DC-based immunotherapy remains controversial. Five clinical trials of DC-based immunotherapy demonstrated that MGMT methylation was an indicator of favorable prognosis in GBM patients [4, 20–24]; however, two trials presented contrasting results [25, 26]. Further research is therefore needed.

In conclusion, we showed the promising activity of TFDC-based immunotherapy in *IDH-WT* GBM and the association of low HLA-A expression and the absence of *CCDC88A*, *KRT4*, *TACC2*, and *TONSL* mutations in tumor cells of patients showing better prognosis. These findings can inform the selection of patients who will clinically benefit from TFDC-based immunotherapy, maximizing favorable prognosis and cost-effectiveness. Expanding upon additional aspects, exploring the correlation between (immunologic) TME under immune-supportive/suppressive conditions and the effectiveness of TFDC immunotherapy presents intriguing subjects for inquiry. We will develop further research to provide proper immune-monitoring which should encompass not only TILs, but also TAMs and myeloid-derived suppressor cells.

## Supplementary Information

Below is the link to the electronic supplementary material.

Supplementary file1 (DOCX 16 KB)

Supplementary file2 (DOCX 22 KB)

Supplementary file3 (DOCX 16 KB)

Supplementary file4 (DOCX 24 KB)

Supplementary file5 (DOCX 18 KB)

Supplementary file6 (DOCX 16 KB)

Supplementary file7 (DOCX 18 KB)

Supplementary file8 (PDF 427 KB)

Supplementary file9 (PDF 448 KB)

Supplementary file10 (PDF 447 KB)

Supplementary file11 (PDF 674 KB)

## Data Availability

The datasets generated during and/or analyzed during the current study are available from the corresponding author on reasonable request.

## Abbreviations

CGGA: Chinese Glioma Genome Atlas
DC: Dendritic cell
DEGs: Differentially expressed genes
FFPE: Formalin-fixed paraffin-embedded
GBM: Glioblastoma
GO: Gene Ontology
ICIs: Immune checkpoint inhibitors
IDH: Isocitrate dehydrogenase
IQR: Interquartile range
KPS: Karnofsky Performance Scale
MGMT: *O*^6^-Methylguanine-DNA methyltransferase
MHC: Major histocompatibility complex
OS: Overall survival
PBMCs: Peripheral blood mononuclear cells
SD: Standard deviation
TAAs: Tumor-associated antigens
TAM: Tumor-associated macrophage
TFDC: Tumor-fused dendritic cell
TIL: Tumor-infiltrating lymphocyte
TMB: Tumor mutational burden
TME: Tumor microenvironment
TMZ: Temozolomide
WHO: World Health Organization

## References

[1] Ostrom QT, Gittleman H, Liao P (2017). CBTRUS statistical report: primary brain and other central nervous system tumors diagnosed in the United States in 2010–2014. Neuro Oncol.

[2] Stupp R, Mason WP, van den Bent MJ (2005). Radiotherapy plus concomitant and adjuvant temozolomide for glioblastoma. N Engl J Med.

[3] Yang K, Wu Z, Zhang H (2022). Glioma targeted therapy: insight into future of molecular approaches. Mol Cancer.

[4] Liau LM, Ashkan K, Brem S (2023). Association of autologous tumor lysate-loaded dendritic cell vaccination with extension of survival among patients with newly diagnosed and recurrent glioblastoma: a phase 3 prospective externally controlled cohort trial. JAMA Oncol.

[5] Steinman RM, Nussenzweig MC (1980). Dendritic cells: features and functions. Immunol Rev.

[6] Schraml BU, Reis e Sousa C (2015). Defining dendritic cells. Curr Opin Immunol.

[7] Huang AY, Golumbek P, Ahmadzadeh M (1994). Role of bone marrow-derived cells in presenting MHC class I-restricted tumor antigens. Science.

[8] Datsi A, Sorg RV (2021). Dendritic cell vaccination of glioblastoma: road to success or dead end. Front Immunol.

[9] Kikuchi T, Akasaki Y, Irie M (2001). Results of a phase I clinical trial of vaccination of glioma patients with fusions of dendritic and glioma cells. Cancer Immunol Immunother.

[10] Kikuchi T, Akasaki Y, Abe T (2004). Vaccination of glioma patients with fusions of dendritic and glioma cells and recombinant human interleukin 12. J Immunother.

[11] Akasaki Y, Kikuchi T, Homma S (2016). Phase I/II trial of combination of temozolomide chemotherapy and immunotherapy with fusions of dendritic and glioma cells in patients with glioblastoma. Cancer Immunol Immunother.

[12] Akasaki Y, Kikuchi T, Irie M (2011). Cotransfection of poly(I: C) and siRNA of IL-10 into fusions of dendritic and glioma cells enhances antitumor T helper type 1 induction in patients with glioma. J Immunother.

[13] Koido S (2016). Dendritic-tumor fusion cell-based cancer vaccines. Int J Mol Sci.

[14] Hao C, Chen G, Zhao H (2020). PD-L1 expression in glioblastoma, the clinical and prognostic significance: a systematic literature review and meta-analysis. Front Oncol.

[15] Yang T, Kong Z, Ma W (2021). PD-1/PD-L1 immune checkpoint inhibitors in glioblastoma: clinical studies, challenges and potential. Hum Vaccin Immunother.

[16] Prins RM, Soto H, Konkankit V (2011). Gene expression profile correlates with T-cell infiltration and relative survival in glioblastoma patients vaccinated with dendritic cell immunotherapy. Clin Cancer Res.

[17] Gromeier M, Brown MC, Zhang G (2021). Very low mutation burden is a feature of inflamed recurrent glioblastomas responsive to cancer immunotherapy. Nat Commun.

[18] Schaafsma E, Fugle CM, Wang X, Cheng C (2021). Pan-cancer association of HLA gene expression with cancer prognosis and immunotherapy efficacy. Br J Cancer.

[19] Zhang H, Zhang N, Wu W (2022). Machine learning-based tumor-infiltrating immune cell-associated lncRNAs for predicting prognosis and immunotherapy response in patients with glioblastoma. Brief Bioinform.

[20] Ardon H, Van Gool SW, Verschuere T (2012). Integration of autologous dendritic cell-based immunotherapy in the standard of care treatment for patients with newly diagnosed glioblastoma: results of the HGG-2006 phase I/II trial. Cancer Immunol Immunother.

[21] Inogés S, Tejada S, de Cerio AL-D (2017). A phase II trial of autologous dendritic cell vaccination and radiochemotherapy following fluorescence-guided surgery in newly diagnosed glioblastoma patients. J Transl Med.

[22] Buchroithner J, Erhart F, Pichler J (2018). Audencel immunotherapy based on dendritic cells has no effect on overall and progression-free survival in newly diagnosed glioblastoma: a phase II randomized trial. Cancers.

[23] Liau LM, Ashkan K, Tran DD (2018). First results on survival from a large phase 3 clinical trial of an autologous dendritic cell vaccine in newly diagnosed glioblastoma. J Transl Med.

[24] Pellegatta S, Eoli M, Cuccarini V (2018). Survival gain in glioblastoma patients treated with dendritic cell immunotherapy is associated with increased NK but not CD8+ T cell activation in the presence of adjuvant temozolomide. Oncoimmunology.

[25] Jan C-I, Tsai W-C, Harn H-J (2018). Predictors of response to autologous dendritic cell therapy in glioblastoma multiforme. Front Immunol.

[26] Hu JL, Omofoye OA, Rudnick JD (2022). A phase I study of autologous dendritic cell vaccine pulsed with allogeneic stem-like cell line lysate in patients with newly diagnosed or recurrent glioblastoma. Clin Cancer Res.

[27] Yamanaka R, Homma J, Yajima N (2005). Clinical evaluation of dendritic cell vaccination for patients with recurrent glioma: results of a clinical phase I/II trial. Clin Cancer Res.

[28] Erhart F, Buchroithner J, Reitermaier R (2018). Immunological analysis of phase II glioblastoma dendritic cell vaccine (Audencel) trial: immune system characteristics influence outcome and Audencel up-regulates Th1-related immunovariables. Acta Neuropathol Commun.

[29] Prins RM, Wang X, Soto H (2013). Comparison of glioma-associated antigen peptide-loaded versus autologous tumor lysate-loaded dendritic cell vaccination in malignant glioma patients. J Immunother.

[30] Louis DN, Giannini C, Capper D (2018). cIMPACT-NOW update 2: diagnostic clarifications for diffuse midline glioma, H3 K27M-mutant and diffuse astrocytoma/anaplastic astrocytoma, IDH-mutant. Acta Neuropathol.

[31] Tamura R, Tanaka T, Ohara K (2019). Persistent restoration to the immunosupportive tumor microenvironment in glioblastoma by bevacizumab. Cancer Sci.

[32] Schindelin J, Arganda-Carreras I, Frise E (2012). Fiji: an open-source platform for biological-image analysis. Nat Methods.

[33] Yatabe Y, Hida T, Horio Y (2006). A rapid, sensitive assay to detect EGFR mutation in small biopsy specimens from lung cancer. J Mol Diagn.

[34] Horbinski C, Kofler J, Kelly LM (2009). Diagnostic use of IDH1/2 mutation analysis in routine clinical testing of formalin-fixed, paraffin-embedded glioma tissues. J Neuropathol Exp Neurol.

[35] Lorente A, Mueller W, Urdangarín E (2008). Detection of methylation in promoter sequences by melting curve analysis-based semiquantitative real time PCR. BMC Cancer.

[36] Bowman RL, Wang Q, Carro A (2017). GlioVis data portal for visualization and analysis of brain tumor expression datasets. Neuro Oncol.

[37] Mayakonda A, Lin D-C, Assenov Y (2018). Maftools: efficient and comprehensive analysis of somatic variants in cancer. Genome Res.

[38] Cyriac Kandoth mskcc/vcf2maf: vcf2maf v1.6.19. (2020). 10.5281/zenodo.593251

[39] McLaren W, Gil L, Hunt SE (2016). The ensembl variant effect predictor. Genome Biol.

[40] Hegi ME, Diserens A-C, Gorlia T (2005). MGMT gene silencing and benefit from temozolomide in glioblastoma. N Engl J Med.

[41] Dhodapkar MV, Steinman RM, Sapp M (1999). Rapid generation of broad T-cell immunity in humans after a single injection of mature dendritic cells. J Clin Invest.

[42] Wang X, Lu J, Guo G, Yu J (2021). Immunotherapy for recurrent glioblastoma: practical insights and challenging prospects. Cell Death Dis.

[43] Yu G, Pang Y, Merchant M (2021). Tumor mutation burden, expressed neoantigens and the immune microenvironment in diffuse gliomas. Cancers.

