# Supplementary material for: Prognostic survival biomarkers of tumor-fused dendritic cell vaccine therapy in patients with newly diagnosed glioblastoma
Source: Cancer Immunol Immunother. 2023 Jun 29;72(10):3175–89. doi: 10.1007/s00262-023-03482-8 (PMC10491709; doi:10.1007/s00262-023-03482-8)
Supplement: Supplementary file 1 — Supplementary file1 (DOCX 16 KB) [file 262_2023_3482_MOESM1_ESM.docx]

| Supplementary Table 1: Cox regression analysis for overall survival in patients with GBM IDH wild-type treated with TFDCs immunotherapy. | | | | |
| --- | --- | --- | --- | --- |
| Univariate |  |  |  |  |
| independent variables | hazard ratio | 95％ confidence interval | p value |  |
| Number of injections | 0.96 | 0.82 to 1.13 | 0.632 |  |
| Number of total dendritic cells (per 1*10^6 cells) | 1.00 | 0.96 to 1.04 | 0.996 |  |
| Number of total cancer cells (per 1*10^5 cells) | 1.00 | 0.99 to 1.01 | 0.672 |  |
| Fusion rates | 1.00 | 0.96 to 1.04 | 0.820 |  |
