# Supplementary material for: Prognostic survival biomarkers of tumor-fused dendritic cell vaccine therapy in patients with newly diagnosed glioblastoma
Source: Cancer Immunol Immunother. 2023 Jun 29;72(10):3175–89. doi: 10.1007/s00262-023-03482-8 (PMC10491709; doi:10.1007/s00262-023-03482-8)
Supplement: Supplementary file 2 — Supplementary file2 (DOCX 22 KB) [file 262_2023_3482_MOESM2_ESM.docx]

| Supplementary Table 2. Characteristics of patients with GBM IDH wild-type divided into two groups based on median survival time. | | | | | |
| --- | --- | --- | --- | --- | --- |
|  |  | short survival | long survival | Total | P-value |
| Numbers |  | 8 | 7 | 15 |  |
| Sex |  |  |  |  | p=0.282* |
| Female |  | 4 | 1 | 5 |  |
| Male |  | 4 | 6 | 10 |  |
| Age–years  Mean ± SD |  | 61.5±9.0 | 53.9±13.8 | 57.9±11.7 | p=0.358† |
| Extent of resection |  |  |  |  | p=0.706* |
| Total |  | 3 | 4 | 7 |  |
| Subtotal |  | 1 | 2 | 3 |  |
| Partial |  | 3 | 1 | 4 |  |
| Biopsy |  | 1 | 0 | 1 |  |
| MGMT profile (MS-PCR) |  |  |  |  | p=0.413* |
| Methylated |  | 4 | 2 | 6 |  |
| Unmethylated |  | 4 | 3 | 7 |  |
| Not detected |  | 0 | 2 | 2 |  |
| Bevacizumab usage |  |  |  |  | p=1.000* |
| yes |  | 3 | 3 | 6 |  |
| no |  | 5 | 4 | 9 |  |
| Preoperative KPS |  |  |  |  | p=0.367† |
| median |  | 80 | 100 | 80 |  |
| IQR |  | 65-90 | 60-100 | 60-100 |  |
| Postoperative KPS |  |  |  |  | p=0.462† |
| median |  | 85 | 100 | 90 |  |
| IQR |  | 65-100 | 70-100 | 70-100 |  |
| RPA2011 classification |  |  |  |  | p=0.765* |
| III |  | 0 | 1 | 1 |  |
| IV |  | 6 | 4 | 10 |  |
| V |  | 2 | 2 | 4 |  |
| Overall survival from vaccination–months |  |  |  |  | p=0.0002†† |
| median |  | 9 | 32 | 16 |  |
| 2year survival rate(%) |  | 0% | 71.4% | 33.3% |  |
| Overall survival – months |  |  |  |  | p=0.0001†† |
| median |  | 18.3 | 40 | 23.3 |  |
| 2year survival rate(%) |  | 0% | 100% | 46.7% |  |
| *Fisher’s exact test, †Mann-Whitney U test, IQR: interquartile range, KPS: Karnofsky performance status  ††log-rank test, RPA: recursive partitioning analysis, MGMT: O6-methylguanine-DNA methyltransferase, MS-PCR: methylation specific-polymerase chain reaction, SD: standard deviation. | | | | | |
