# Supplementary material for: Prognostic survival biomarkers of tumor-fused dendritic cell vaccine therapy in patients with newly diagnosed glioblastoma
Source: Cancer Immunol Immunother. 2023 Jun 29;72(10):3175–89. doi: 10.1007/s00262-023-03482-8 (PMC10491709; doi:10.1007/s00262-023-03482-8)
Supplement: Supplementary file 3 — Supplementary file3 (DOCX 16 KB) [file 262_2023_3482_MOESM3_ESM.docx]

| Supplementary Table 3: Cox regression analysis for overall survival. | | | |
| --- | --- | --- | --- |
| Univariate |  |  |  |
| independent variables | hazard ratio | 95％ confidence interval | p-value |
| HLA-A | 4.63 | 1.20 – 17.9 | 0.026 |
| HLA-B | 0.84 | 0.27 – 2.64 | 0.771 |
| HLA-C | 0.84 | 0.27 – 2.64 | 0.771 |
| HLA-DPA | 2.15 | 0.67 – 6.90 | 0.198 |
| HLA-DQA | 0.42 | 0.13 – 1.34 | 0.143 |
| HLA-DRA | 0.60 | 0.19 – 1.88 | 0.380 |
