# Supplementary material for: Prognostic survival biomarkers of tumor-fused dendritic cell vaccine therapy in patients with newly diagnosed glioblastoma
Source: Cancer Immunol Immunother. 2023 Jun 29;72(10):3175–89. doi: 10.1007/s00262-023-03482-8 (PMC10491709; doi:10.1007/s00262-023-03482-8)
Supplement: Supplementary file 4 — Supplementary file4 (DOCX 24 KB) [file 262_2023_3482_MOESM4_ESM.docx]

| Supplementary Table 4: Cox regression analysis for overall survival. | | | |
| --- | --- | --- | --- |
| Univariate |  |  |  |
| independent variables | hazard ratio | 95％ confidence interval | p-value |
| ACTA1 | 0.70 | 0.15 – 3.28 | 0.648 |
| ADAMTS14 | 1.82 | 0.47 – 7.10 | 0.388 |
| AHNAK2 | 3.66 | 0.81 – 16.6 | 0.093 |
| AMN | 2.35 | 0.65 – 8.51 | 0.192 |
| APC2 | 0.82 | 0.18 – 3.80 | 0.796 |
| C2orf71 | 0.65 | 0.14 – 3.04 | 0.589 |
| CCDC88A | 8.76 | 1.39 – 55.1 | 0.021 |
| COL6A3 | 2.06 | 0.62 – 6.84 | 0.240 |
| DHX34 | 0.25 | 0.03 – 1.99 | 0.190 |
| DHX57 | 1.89 | 0.47 – 7.69 | 0.371 |
| DNAH17 | 0.67 | 0.14 – 3.16 | 0.614 |
| DNAH3 | 0.95 | 0.20 – 4.43 | 0.949 |
| DNHD1 | 0.52 | 0.14 – 1.96 | 0.333 |
| ETFDH | 0.59 | 0.12 – 2.81 | 0.505 |
| FSIP2 | 0.82 | 0.18 – 3.80 | 0.796 |
| GSE1 | 0.65 | 0.17 – 2.48 | 0.525 |
| IRF5 | 1.26 | 0.38 – 4.18 | 0.705 |
| KMT2B | 2.44 | 0.58 – 10.3 | 0.225 |
| KRT4 | 4.58 | 0.99 – 21.1 | 0.051 |
| KRTAP4-7 | 3.62 | 0.84 – 15.6 | 0.083 |
| LCE4A | 2.26 | 0.64 – 7.95 | 0.203 |
| LRP5L | 0.27 | 0.03 – 2.13 | 0.212 |
| LRRK1 | 3.66 | 0.81 – 16.6 | 0.093 |
| MAST4 | 2.40 | 0.61 – 9.48 | 0.210 |
| MUC16 | 1.15 | 0.24 – 5.41 | 0.862 |
| MYBPC2 | 1.62 | 0.42 – 6.31 | 0.487 |
| MYH9 | 1.15 | 0.30 – 4.40 | 0.841 |
| OBSCN | 2.44 | 0.58 – 10.3 | 0.225 |
| OR2B2 | 1.02 | 0.29 – 3.54 | 0.978 |
| OTOL1 | 0.45 | 0.10 – 2.09 | 0.307 |
| POGK | 1.39 | 0.36 – 5.30 | 0.630 |
| POMZP3 | 1.13 | 0.30 – 4.29 | 0.860 |
| PREX2 | 0.59 | 0.12 – 2.81 | 0.505 |
| PTBP2 | 0.18 | 0.23 – 1.44 | 0.107 |
| RP11-1055B8.7 | 0.82 | 0.18 – 3.80 | 0.796 |
| RTL1 | 1.38 | 0.36 – 5.23 | 0.639 |
| SFSWAP | 0.72 | 0.15 – 3.33 | 0.670 |
| SIPA1L3 | 0.72 | 0.15 – 3.33 | 0.670 |
| STEAP2 | 0.16 | 0.20 – 1.30 | 0.087 |
| SYNE1 | 1.18 | 0.35 – 3.89 | 0.792 |
| SYNE2 | 0.88 | 0.19 – 4.11 | 0.876 |
| TACC2 | 9.62 | 1.55 – 59.6 | 0.015 |
| TECTA | 0.27 | 0.03 – 2.13 | 0.212 |
| TENM3 | 0.82 | 0.18 – 3.80 | 0.796 |
| TEX2 | 1.29 | 0.27 – 6.07 | 0.751 |
| THBS2 | 1.08 | 0.22 – 4.75 | 0.982 |
| TINAG | 1.73 | 0.45 – 6.59 | 0.421 |
| TMEM131 | 0.86 | 0.19 – 4.08 | 0.858 |
| TONSL | 3.60 | 0.94 – 13.8 | 0.061 |
| TP53 | 0.67 | 0.14 – 3.16 | 0.614 |
| TTN | 0.72 | 0.21 – 2.49 | 0.604 |
| WDR81 | 0.65 | 0.14 – 3.03 | 0.579 |
| ZC3H10 | 2.49 | 0.62 – 10.1 | 0.201 |
| ZNF717 | 0.75 | 0.16 – 3.48 | 0.710 |
