# Supplementary material for: Prognostic survival biomarkers of tumor-fused dendritic cell vaccine therapy in patients with newly diagnosed glioblastoma
Source: Cancer Immunol Immunother. 2023 Jun 29;72(10):3175–89. doi: 10.1007/s00262-023-03482-8 (PMC10491709; doi:10.1007/s00262-023-03482-8)
Supplement: Supplementary file 6 — Supplementary file6 (DOCX 16 KB) [file 262_2023_3482_MOESM6_ESM.docx]

| Supplementary Table 6: Logrank test for overall survival. | | |
| --- | --- | --- |
| independent variables |  | p-value |
| AHNAK2 |  | 0.0680 |
| CCDC88A |  | 0.0056 |
| KRT4 |  | 0.0317 |
| KRTAP4-7 |  | 0.0627 |
| LRRK1 |  | 0.0680 |
| STEAP2 |  | 0.0512 |
| TACC2 |  | 0.0029 |
| TONSL |  | 0.0442 |
