# Supplementary material for: Prognostic survival biomarkers of tumor-fused dendritic cell vaccine therapy in patients with newly diagnosed glioblastoma
Source: Cancer Immunol Immunother. 2023 Jun 29;72(10):3175–89. doi: 10.1007/s00262-023-03482-8 (PMC10491709; doi:10.1007/s00262-023-03482-8)
Supplement: Supplementary file 7 — Supplementary file7 (DOCX 18 KB) [file 262_2023_3482_MOESM7_ESM.docx]

| Supplementary Table 7: Genetic variants of patients with GBM IDH wild-type stratified by MGMT promoter methylation. | | | | | |
| --- | --- | --- | --- | --- | --- |
|  |  | MGMT unmethylated | MGMT methylated | Not detected | P-value |
| Numbers |  | 6 | 6 | 2 |  |
| CCDC88A |  |  |  |  | p=0.143* |
| Wild |  | 6 | 3 | 2 |  |
| Mutant |  | 0 | 3 | 0 |  |
| KRT4 |  |  |  |  | p=1.000* |
| Wild |  | 5 | 4 | 2 |  |
| Mutant |  | 1 | 2 | 0 |  |
| TACC2 |  |  |  |  | p=1.000* |
| Wild |  | 5 | 4 | 2 |  |
| Mutant |  | 1 | 2 | 0 |  |
| TONSL |  |  |  |  | p=1.000* |
| Wild |  | 4 | 4 | 1 |  |
| Mutant |  | 2 | 2 | 1 |  |
|  |  |  |  |  |  |
| *Fisher’s exact test, GBM: glioblastoma, IDH:isocitrate dehydrogenase, MGMT: O6-methylguanine-DNA methyltransferase. | | | | | |
