# Supplementary material for: Prognostic survival biomarkers of tumor-fused dendritic cell vaccine therapy in patients with newly diagnosed glioblastoma
Source: Cancer Immunol Immunother. 2023 Jun 29;72(10):3175–89. doi: 10.1007/s00262-023-03482-8 (PMC10491709; doi:10.1007/s00262-023-03482-8)
Supplement: Supplementary file 8 — Supplementary file8 (PDF 427 KB) [file 262_2023_3482_MOESM8_ESM.pdf]

Supplemental Figure 1

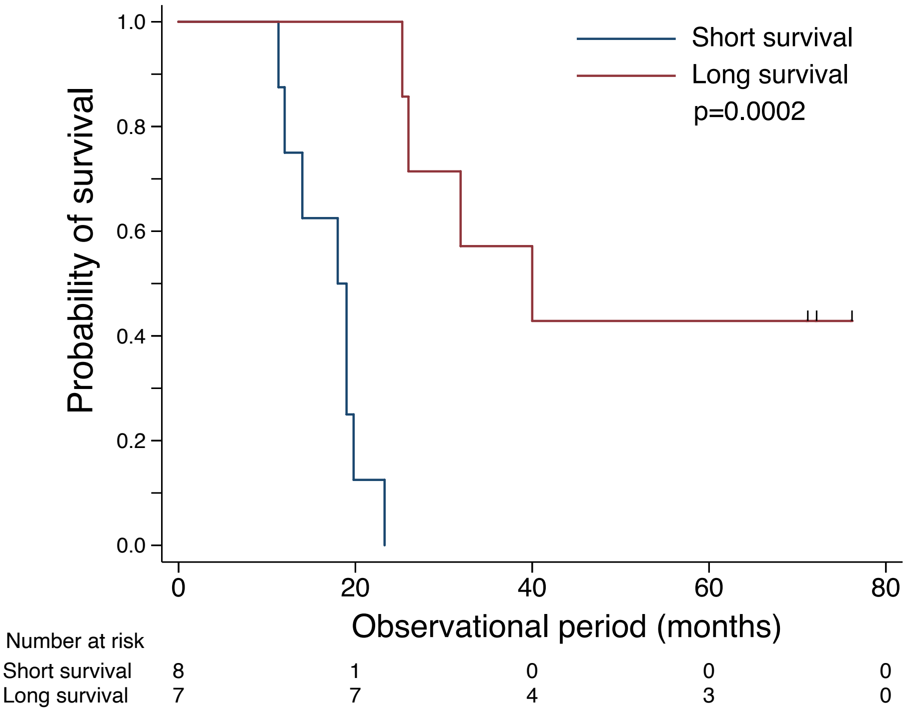

Supplementary Fig. 1 Kaplan-Meier analysis showing the probability of survival in GBM IDH wild-type tumors stratified by median OS (n=15). Kaplan–Meier survival curves for patients with long- and short-survival times in the study cohort
