# Supplementary material for: Prognostic survival biomarkers of tumor-fused dendritic cell vaccine therapy in patients with newly diagnosed glioblastoma
Source: Cancer Immunol Immunother. 2023 Jun 29;72(10):3175–89. doi: 10.1007/s00262-023-03482-8 (PMC10491709; doi:10.1007/s00262-023-03482-8)
Supplement: Supplementary file 9 — Supplementary file9 (PDF 448 KB) [file 262_2023_3482_MOESM9_ESM.pdf]

Supplemental Figure 2

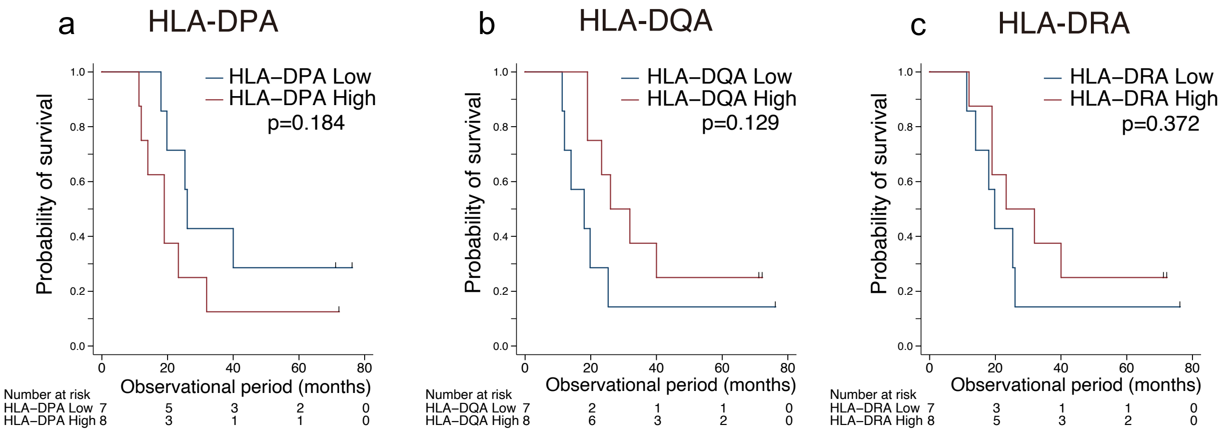

Supplementary Fig. 2 Kaplan-Meier analysis showing the probability of survival in GBM IDH wild-type tumors stratified by HLA Class II gene expression (n=15). a-c Kaplan–Meier survival curves for patients with high and low expression levels of HLA-DPA (a), HLA-DQA (b), and HLA-DRA (c) in the study cohort
