# Supplementary material for: Prognostic survival biomarkers of tumor-fused dendritic cell vaccine therapy in patients with newly diagnosed glioblastoma
Source: Cancer Immunol Immunother. 2023 Jun 29;72(10):3175–89. doi: 10.1007/s00262-023-03482-8 (PMC10491709; doi:10.1007/s00262-023-03482-8)
Supplement: Supplementary file 10 — Supplementary file10 (PDF 447 KB) [file 262_2023_3482_MOESM10_ESM.pdf]

Supplemental Figure 3

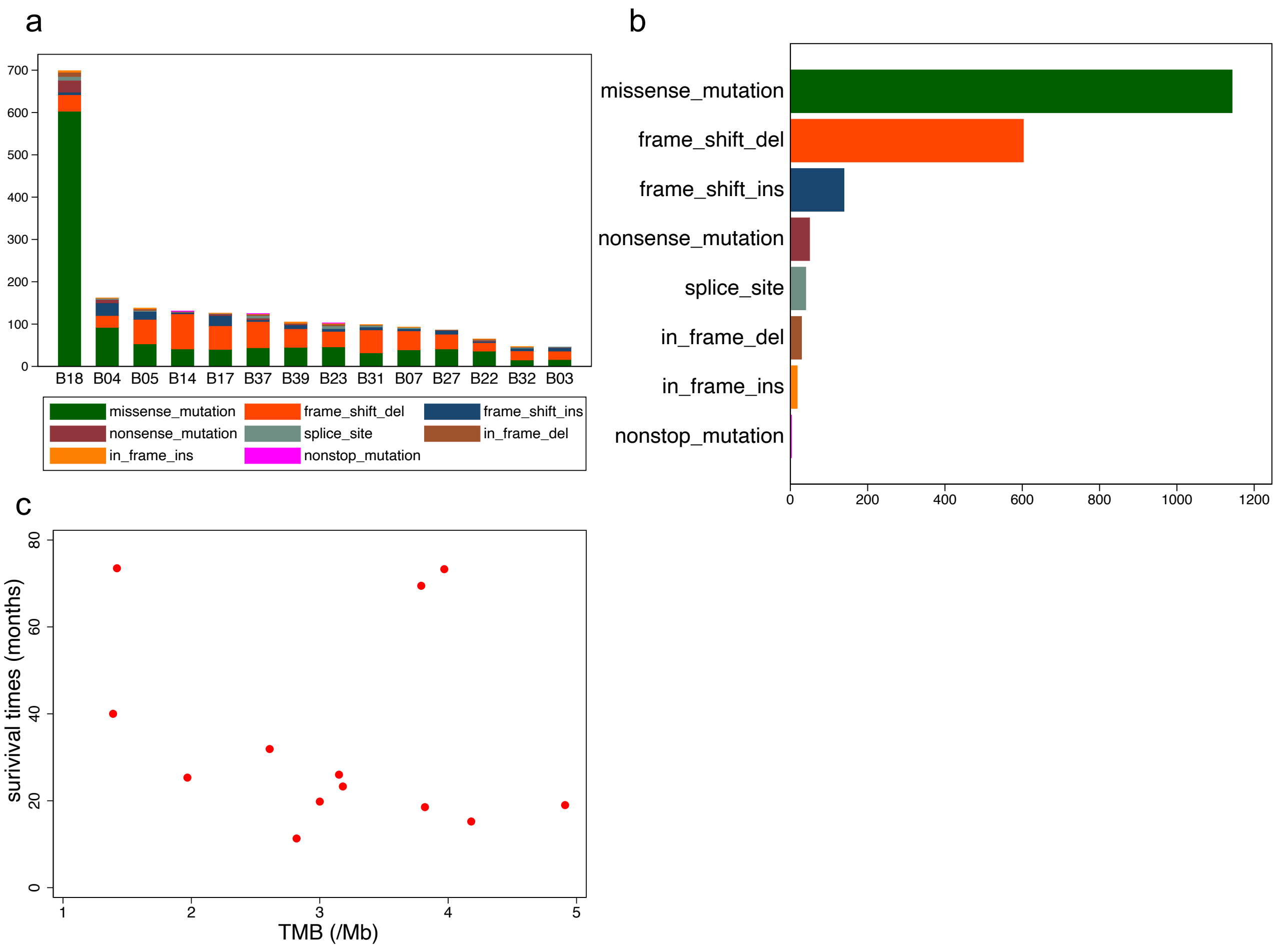

Supplementary Fig. 3 Whole exome analysis in tumor cells (n=14). **a** Bar plot showing genetic variants per tumor cell. **b** Bar plot showing the classification of genetic variants. **c** Scatter plot of survival times and TMB (n=13) and the outlier (B18) was excluded
