# Supplementary material for: Prognostic survival biomarkers of tumor-fused dendritic cell vaccine therapy in patients with newly diagnosed glioblastoma
Source: Cancer Immunol Immunother. 2023 Jun 29;72(10):3175–89. doi: 10.1007/s00262-023-03482-8 (PMC10491709; doi:10.1007/s00262-023-03482-8)
Supplement: Supplementary file 11 — Supplementary file11 (PDF 674 KB) [file 262_2023_3482_MOESM11_ESM.pdf]

Supplemental Figure 4

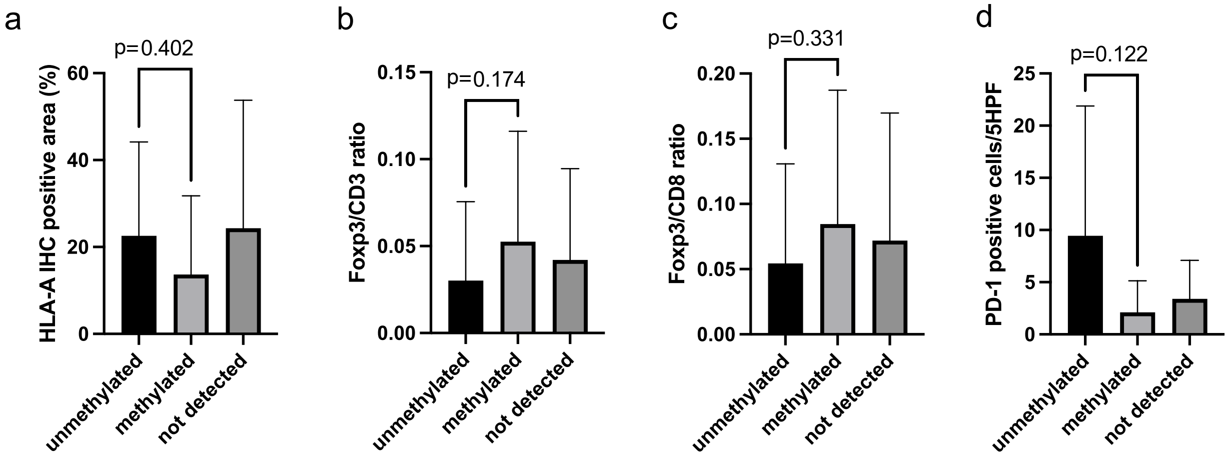

Supplementary Fig. 4 Bar plot showing the HLA expression in tumor cells and immunoregulatory cell infiltration in the tumor stratified by MGMT promoter methylation status. Data are presented as the mean ± standard deviation (SD). **a** HLA-A IHC-positive areas were compared between unmethylated (n = 9), methylated (n = 14), and not detected (n = 5) groups. **b, c** Foxp3/CD3- (**b**) and Foxp3/CD8- (**c**) positive cell ratios in the unmethylated (n = 9), methylated (n = 14), and not detected (n = 5) groups. **d** Numbers of PD-1-positive cells in the unmethylated (n = 9), methylated (n = 14), and not detected (n = 5) groups
